# Determinants of Exclusive Breastfeeding of Infants under Six Months among Cambodian Mothers

**DOI:** 10.1155/2020/2097285

**Published:** 2020-08-24

**Authors:** Sopheak Um, Ying Zhen Charissa Chan, Bunkea Tol, Heng Sopheab

**Affiliations:** ^1^The School of Public Health at the National Institute of Public Health, Phnom Penh, Cambodia; ^2^The University of Melbourne, Parkville, Victoria 3010, Australia

## Abstract

**Introduction:**

Practicing exclusive breastfeeding (EBF) in an infant's first six months of life is recommended by the World Health Organization because of its proven effectiveness as a method to support the infant's short- and long-term physical and cognitive development. However, many countries, including Cambodia, face contextually driven challenges in meeting this optimum standard of breastfeeding. The recent declining EBF rate in Cambodia is a concerning indicator of the impact of these challenges.

**Methods:**

We used existing data from the 2014 Cambodian Demographic and Health Survey (CDHS) to analyze 717 Cambodian mother-infant pairs. CDHS 2014 used a two-stage stratified cluster sampling approach to select samples. A multivariable logistic regression analysis was used to assess determinants of EBF, taking into account the sampling weight in the analysis. Adjusted odds ratios (AOR) with 95% confidence intervals (CI) were calculated, and significance level was considered at *p* value < 0.05.

**Results:**

Our findings indicate that among mothers with infants under six months, EBF was more likely if they resided rurally (AOR = 2.28; 95% CI 1.23-4.23) and if they delivered at a public hospital (AOR = 2.64; 95% CI 1.28-5.47). On the other hand, mothers of middle wealth index practiced EBF less than mothers of low wealth index (AOR = 0.58; 95% CI 0.34-0.99). And as expected, our analysis confirmed that the older the infants grew, the less likely they were to be exclusively breastfed than those younger than one month old (2-3 months: AOR = 0.49; 95% CI 0.26-0.92; 4-5 months: AOR = 0.25; 95% CI 0.15-0.43).

**Conclusion:**

The findings emphasize the need to address these determinants adequately by appropriate interventions to halt the declining trend of EBF practice. We recommend a multifaceted approach to improve EBF rates in Cambodia. Advocacy around EBF at public hospitals should continue, and private hospital staff should receive training to provide EBF counselling and support to mothers.

## 1. Introduction

The early initiation and continuation of exclusive breastfeeding (EBF) is widely acknowledged as necessary to reduce infection-related mortality and morbidity in infants and is associated with better long-term nutritional and cognitive outcomes in the child [1–4]. The World Health Organization (WHO) recommends EBF for the first six months of life to achieve optimal outcomes [1]. EBF entails feeding the infant strictly with breastmilk, excluding all other liquid, solid, or semisolid foods, which include water and breastmilk substitutes. For the breastfeeding mother, lactational amenorrhea is an effective and economical contraceptive method. There is also evidence that EBF reduces a mother's long-term risk of developing breast and ovarian cancer [5, 6].

The prevalence of EBF among Cambodian mothers with infants under six months, as found in the 2014 Cambodian Demographic and Health Survey (CDHS), is 65.2% [7]. This meets the WHO's global nutrition target of increasing the rate of EBF in the first six months of life to at least 50%, so to achieve the Sustainable Development Goal (SDG) Target 2.2 to end malnutrition by 2030 [8]. The prevalence of EBF in Cambodia is higher than many other developing countries, as found in studies conducted in Ethiopia in 2015 (29.3%), Indonesia in 2012 (43.6%), Sri Lanka in 2016 (50.8%), Bangladesh in 2014 (55%), and Ghana in 2017 (63%) [9–13]. Cambodia also fared better in EBF practice than some developed countries like the United Arab Emirates (25%) and Australia (15.4%) [14, 15]. Cambodia's better relative performance is in addition to a reduction in infant mortality rates between 2010 and 2014 [7, 16]. However, there has in fact been a decline in the practice of EBF by 8.3% from 2010 [7, 16]. The WHO recommends that countries which have met the target of at least 50% should strive for an increase in EBF of at least 1.2% each year [17]. This downward trend in EBF that Cambodia faces is not only in opposition to the recommendation of increasing EBF rates but may also derail Cambodia from maintaining the WHO's SDG target of EBF rate.

These declining EBF rates may be partly influenced by an increasing use of breastmilk substitutes, in the context of pervasive promotion of breastmilk substitutes in Cambodia [18, 19]. This phenomenon has been identified in other countries like Laos and Senegal. [20, 21] However, the presence of breastmilk substitutes is just one factor and has been a longstanding issue. It does not comprehensively explain the recent trend in EBF rates. There is still paucity in more holistic evidence around what factors determine maternal practice and uptake of EBF in their infant's first six months of life. Our study analyzed maternal sociodemographic and health behavioral factors to identify possible determinants of EBF among Cambodian mothers. In doing so, our results may guide the shaping of public health policy to reverse the current trend of EBF practice.

## 2. Materials and Methods

### 2.1. Data Source

This study used existing data from the 2014 CDHS, a nationally representative cross-sectional study design. The survey collected samples from 19 sampling domains in Cambodia including Banteay Meanchey, Battambang-Pailin, Kampong Cham, Kampong Chhnang, Kampong Speu, Kampong Thom, Kampot-Kep, Kandal, Kratie, Mondul Kiri-Ratanak Kiri, Otdar Meanchey, Phnom Penh, Preah Sihanouk-Koh Kong, Preah Vihear-Stung Treng, Prey Veng, Pursat, Siem Reap, Svay Rieng, and Takeo. The 2014 CDHS study adopted a two-stage stratified cluster sampling method where the sample was divided into 38 sample strata between urban and rural populations. The first sampling stage involved randomly selected clusters of 423 rural enumeration areas (EA) and 188 urban EAs with probability proportional to EA size from 24,210 rural EAs and 4,245 urban EAs, respectively. In the second stage, 11,844 rural households and 4,512 urban households were selected from their respective clusters. A systematic random sampling method was then used to select 24 households per urban cluster and 28 households per rural cluster. All identified women, aged 15-49 years, from the selected households were invited for the interview.

**Figure 1:**
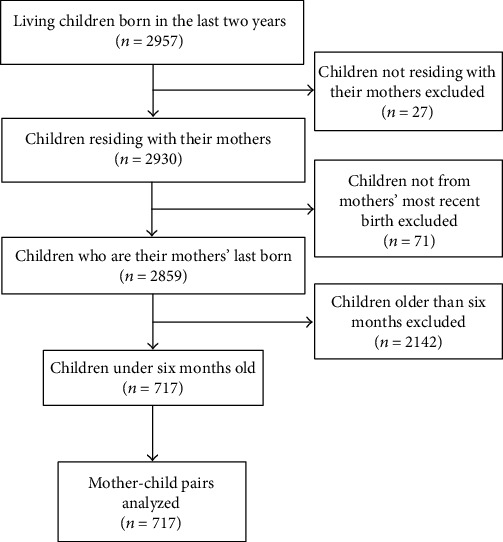
Flow of sample selection based on CDHS 2014 data.

Of these mothers, a subsequent total of 2,957 living children born in the last two years were identified. After accounting for our inclusion and exclusion criteria as indicated in Figure 1, a resultant 717 mother-infant pairs were analyzed.

### 2.2. Dependent Variable

The dependent variable of the study was exclusive breastfeeding within the 24 hours preceding the interview. Those who met this criterion were coded as *yes* (EBF coded 1) and those that did not as *no* (otherwise coded 0).

### 2.3. Independent Variables

The independent variables included in the analysis were classified into sociodemographic characteristics and maternal healthcare access and behavioral factors. The sociodemographic variables were residence (urban, rural), maternal age (<18, 18-34, 35-49), maternal education (no schooling, primary school, secondary school/higher), current marital status (currently married/in union, formerly married), employment status (no job/not working, agricultural/self-employed, professional/technical/sales, others), wealth index (low, middle, high), religion (Buddhist, others), infant birth weight (<2500 grams, ≥2500 grams), infant birth order, sex of infant, infant age, and singleton child status. Wealth index was categorized into *low*, *middle*, and *high*, based on the CDHS 2014 classification of wealth quintiles. Wealth quintiles took into account a family's assets (e.g., radio, television), properties (e.g., farm animals, agricultural land), and durable possessions (e.g., bicycle, motorbike) as a measurement of their wealth—a more accurate assessment of socioeconomic standing and relative affluence in Cambodia as opposed to quantitative income which is often inaccurately reported. Maternal healthcare access and behavioral factors included time of breastfeeding initiation, delivery place (public hospital, private facilities, others), delivery mode, if they received antenatal care (ANC) checks (<4 times, ≥4 times), if they received postnatal care (PNC) checks within the first two months of birth, cigarette smoking, and contraceptive use.

### 2.4. Statistical Analysis

The analysis was performed with Stata 12 (StataCorps, Texas). We analyzed the data by taking into account the sampling weight and complex sampling design of the CDHS data. Descriptive statistical analysis was performed to summarize variables. Frequencies and proportions were calculated for categorical variables, such as occupation, residence, and wealth index. Arithmetic mean and standard deviations (SD) were calculated for all continuous variables, such as mother's age and infant's weight. The bivariable logistic regression analysis was used to assess the relationship between the dependent variable (EBF) and independent variables including sociodemographic variables (i.e., age, residence) and maternal healthcare access and behaviors (i.e., ANC and PNC checks). Covariates that were significantly associated with EBF with a *p* value ≤ 0.10 in the bivariable analysis were included in the multivariable logistic regression to assess the independent effects on the EBF [22, 23]. Also, potential confounding factors were included in the multivariable logistic regression analysis regardless of their significance level, namely, maternal age, maternal education, and whether breastfeeding within the first hour of delivery was initiated. The *p* value < 0.05 was used as a statistically significant level throughout the analysis and expressed in both adjusted odds ratio (AOR) and 95% confidence intervals (CI).

## 3. Ethics

Ethics approval was obtained from the National Ethics Committee for Health Research for the CDHS 2014 (Ref: 056 NECHR), Cambodia, and the Institutional Review Board (IRB) of ICF International in Rockville, Maryland, USA. The publicly available CDHS data was provided to us upon request to the DHS Program, ICF website at https://dhsprogram.com/data/. Written consent was obtained from all participants before the CDHS interview.

## 4. Results

### 4.1. Sociodemographic Characteristics of Mothers and Infants

The mean age of mothers was 26.2 ± 0.4 years, where the large majority were aged between 18 and 34 years old (88.5%). Of the 717 mother-infant pairs, 86% lived in rural areas. Almost half of the mothers' highest education level was primary school (49%). Ninety-seven percent of mothers were either currently married or in union at the time of interview. Forty-two percent of mothers were unemployed, and 27% were in either agricultural or self-employment. Approximately half of the infants were girls (52%). The mean age of infants was 2.7 ± 1.6 months, and almost all infants were of singleton births (98%) (Table 1).

### 4.2. Maternal Healthcare Access and Behaviors

Seventy-four percent of mothers reported delivering at public hospitals. Eleven percent of mothers delivered by cesarean section. We also found that about two-thirds of mothers (64%) started breastfeeding their infant within the first hour of delivery. Eighteen percent of mothers were contraceptive users. Furthermore, 57% of mothers attended at least 4 ANC visits during their pregnancy, and 83% of mothers received PNC check-ups within 2 months of delivery (Table 1).

### 4.3. Factors Associated with EBF of Infants under Six Months

In the final model of the multivariable logistic regression, 10 covariates were included. They were residence, maternal age, maternal education, employment, wealth index, sex of the infant, infant age, delivery place, early breastfeeding, and current contraceptive use. However, only four covariates—residence, wealth index, maternal delivery place, and infant age—were significantly and independently associated with EBF. Mothers who lived in rural areas had higher odds of practicing EBF than urban mothers (AOR = 2.2; 95% CI 1.23-4.18). Mothers who were in the middle wealth quintile were about 42% less likely to breastfeed than mothers in the low wealth quintile (AOR = 0.58; 95% CI 0.34-0.99). It was also noted that as infants grew older, their mothers were less likely to breastfeed them. Infants aged 2-3 months and 4-5 months were exclusively breastfed 51% (AOR = 0.49; 95% CI 0.26-0.92) and 75% (AOR = 0.25; 95% CI 0.15-0.43) less than infants under one month, respectively. Finally, mothers who delivered in public hospitals were more likely to practice EBF than those who delivered in private facilities (AOR = 2.64; 95% CI 1.28-5.47).

However, the following factors were no longer significantly associated with EBF: maternal age, sex of the infant, current use of contraceptive, education level, occupation, and timely initiation of breastfeeding (Table 2).

## 5. Discussion

Overall, our findings show that there were a mix of maternal and infant sociodemographic characteristics and maternal health behaviors positively associated with EBF. These sociodemographic determinants include rural residence, low wealth index, younger infant age, and delivery at a public hospital.

Cambodian mothers living in rural areas were 2.3 times more likely to breastfeed their infants as compared to urban mothers. A study in Malaysia also found that mothers from rural areas were about 20% more likely to exclusively breastfeed than those from urban regions [24]. A possible explanation is that rural mothers may have had less influence and exposure to breastmilk substituents via media marketing than urban mothers who may have mistakenly derived the value of breastmilk substitutes from its modernity [18, 19]. In addition, because of greater job opportunities in urban regions and that Cambodians who value working tend to move from rural to urban areas to live, urban mothers tend to prioritize nondomestic work more than rural mothers [25, 26]. Urban mothers are therefore likely to spend less time domestically than their rural counterparts, leaving them less opportunity to exclusively breastfeed their infants.

This study also showed that mothers who scored low on wealth index were more likely to breastfeed than those who scored middle on wealth index. This difference in socioeconomic status determining breastfeeding practice is consistent with studies conducted in Ethiopia and other East and Southeast Asian countries [9, 27–30]. It can be postulated that because mothers of low wealth index faced greater financial constraints, it removed their power to purchase breastmilk substitutions; thus, they had little choice but to breastfeed their infants exclusively. On the contrary, Anstey et al. reported that the wealthier American mothers were, the more likely they were to breastfeed their child [31]. Suggestions for this phenomenon were that wealthier parents had greater access to infrastructure that supported breastfeeding, such as longer maternity leaves, and jobs that allowed for pumping breaks. Unlike in Cambodia, breastfeeding in America was also part of a desirable status symbol that many parents sought after.

As compared to infants under one month old, those between 2-3 and 4-5 months old were 51% and 75% less likely to be exclusively breastfed, respectively. This means that the older the child was, the less likely they were exclusively breastfed. This result is unsurprising as there are a few known explanations for this trend. Firstly, mothers were more likely to return to work as the child grew older and hence unable to breastfeed their child while away from them. Secondly, the production of breastmilk is dependent on consistent breastfeeding even through the night. Hence, the older the infant, the higher the probability that mothers will be less consistent in keeping up with the demands of breastfeeding. Finally, maternal breastfeeding complications such as mastitis or nipple fissures increase with prolonged breastfeeding, making it increasingly likely for mothers to stop EBF the longer they practiced it. Colombo et al. had found these health complications to be a factor associated with cessation in exclusive breastfeeding practice [2].

An interesting finding from our analysis was that infants delivered in public hospitals were three times more likely to have been exclusively breastfed than those delivered at private facilities. Cambodian private clinics are poorly regulated and monitored, in addition to their financial incentivization by companies to advertise breastmilk substituents to mothers [32]. Hence, they are likely to have inadequate training nor adhered to governmental health recommendations around educating mothers about EBF practice. However, an epidemiological study in Indonesia found no correlation between delivery location and EBF practice [33]. This contrast could suggest that the association of delivery place with EBF practice is contextually driven.

Among the factors not found to be associated with EBF practice was maternal education level. At the time of the study, Cambodia's education system had yet to incorporate maternal and infant health education into its curriculum. This may explain why mothers' EBF practice were determined by other factors apart from education level. Education thus remains a possible area of focus to increase uptake of EBF practice according to WHO guidelines.

There have been interventions trialed to improve the uptake of EBF in Cambodia. A 2016 study found that the involvement of community Buddhist nuns in promoting EBF in addition to the mothers' participation in support groups increased EBF rates [34]. Bazzano et al. conducted a qualitative study in Cambodia and identified the importance of enhancing breastfeeding support through improving the counselling skills of health providers, involving family members, and providing community outreach programs in Cambodia [35].

## 6. Conclusions and Recommendations

While we are unable to draw causality in this study, our results provide context-specific insight into the areas that public health policy may look further into to improve rates of EBF practice among Cambodian mothers. Given our study's findings and proven interventions, we recommend a multifaceted approach to increasing the rates of EBF. Greater surveillance of private hospitals' adherence to EBF recommendations and training around counselling in EBF should be introduced. Public hospitals should continue advocating for EBF during antenatal and postnatal care visits, with emphasis on the importance of maintaining EBF throughout the infant's first six months. In addition, formation of support groups for mothers in the densely populated urban regions of Cambodia may prove beneficial, especially since many mothers come from rural homes and do not have as much family support when in urban regions. Future studies can consider looking in greater depth at maternal perceptions of breastfeeding in Cambodia to understand how these affect breastfeeding behaviors so that interventions can be continually improved accordingly.

## Figures and Tables

**Table 1:** Sociodemographic characteristics and maternal healthcare access and behaviors.

Characteristics	*N* = 717	
	Freq.	%
Residence		
Urban	99	13.8
Rural	618	86.2
Maternal age in years		
<18	33	4.6
18-34	634	**88.5**
35-49	49	6.9
Mean mother age in year (SD)	26.2 (0.4)	
Maternal education (*n* = 716)		
No schooling	81	11.3
Primary school	353	**49.3**
Secondary school/higher	282	39.4
Current marital status		
Currently married/in union	692	96.5
Formerly married	25	3.5
Employment		
No job/not working	297	**41.5**
Agricultural/self-employed	190	26.5
Professional/technical/sales	114	15.9
Others	116	16.1
Wealth index		
Low	296	**41.4**
Middle	152	21.1
High	269	37.5
Religion		
Buddhist	686	95.8
Others	30	4.2
Infant weight at birth (gram)		
≥2500	649	90.6
<2500	67	9.4
Mean infant weight in gram (SD)	3468.7 (1780)	
Birth order		
1^st^	305	42.6
2^nd^	226	31.6
3^rd^	98	13.6
≥4^th^	87	12.2
Sex of infant		
Boy	344	48.0
Girl	373	52.0
Infant age in months (*n* = 716)		
≤1	210	29.4
2–3	257	35.8
4–5	249	34.8
Mean age of infant in month (SD)	2.7 (1.6)	
Singleton child status		
Single birth	704	98.2
Twin or multiple	13	1.8
Exclusive breastfeeding	467	65.2
Early breastfeeding (in hour)		
≥1 hour	253	36.2
<1 hour	445	**63.8**
Maternal delivery place		
Public hospitals	532	**74.3**
Private facilities	127	17.7
Others	57	8.0
Delivery by cesarean section	75	10.5
Report of smoking cigarette	18	2.6
Current contraceptive use	129	17.9
Received at least 4 ANC checks during pregnancy	406	56.6
Received PNC check within two months of delivery	577	**83.3**

**Table 2:** Factors associated with EBF among infants aged less than 6 months.

Variable	Total sample (*N* = 717)				*N* = 698		
	*n*	(% EBF)	COR	95% CI	AOR	95% CI	*p* value
Residence							
Urban	38	38.9	1	Ref.	1	Ref.	
Rural	429	69.4	3.56	**2.20-5.77**	**2.28**	**1.23-4.23**	**0.009**
Maternal age (year)							
<18	18	53.8	1	Ref.	1	Ref.	
18-34	424	66.8	1.73	0.70-4.26	1.9	0.76-4.72	0.167
35-49	26	51.8	0.92	0.29-2.92	1.04	0.31-3.50	0.955
Maternal education							
No schooling	52	63.8	1	Ref.	1	Ref.	
Primary school	243	68.7	1.24	0.66-2.33	1.75	0.84-3.67	0.136
Secondary school/higher	173	61.3	0.9	0.47-1.74	1.94	0.89-4.25	0.097
Employment							
No job/not working	202	67.9	1	Ref.	1	Ref.	
Agriculture/self-employed	139	73.0	1.28	0.75-2.16	1.01	0.55-1.86	0.979
Professional/technical/sale	62	54.2	0.56	**0.32-0.97**	0.77	0.41-1.42	0.395
Others	65	56.4	0.61	0.32-1.15	0.58	0.30-1.13	0.111
Wealth index							
Low	222	75.0	1	**Ref.**	1	Ref.	
Middle	100	66.3	0.66	0.41-1.06	**0.58**	**0.34-0.99**	**0.044**
High	144	53.8	0.39	**0.23-0.65**	0.58	0.30-1.12	0.106
Sex of infant							
Boy	209	60.7	1	Ref.	1	Ref.	
Girl	259	69.3	1.46	**1.02-2.11**	1.48	0.98-2.25	0.062
Infant age (in months)							
≤1	168	79.9	1	Ref.	1	Ref.	
2–3	172	67.1	0.51	**0.27-0.97**	**0.49**	**0.26-0.92**	**0.027**
4–5	127	50.9	0.26	**0.16-0.42**	**0.25**	**0.15-0.43**	**<0.001**
Delivery place							
Private facilities	57	44.9	1	Ref.	1	Ref.	
Public hospital	374	70.3	2.9	**1.53-5.49**	**2.64**	**1.28-5.47**	**0.009**
Others	36	62.9	2.08	0.94-4.64	1.43	0.54-3.80	0.472
Early breastfeeding (hour)							
≥1	159	63.1	1	Ref.	1	Ref.	
<1	308	69.1	1.31	0.87-1.98	1.18	0.71-1.96	0.517
Currently contraceptive use							
No	405	68.8	1	Ref.	1	Ref.	
Yes	63	48.7	0.43	**0.26-0.70**	0.67	0.36-1.22	0.187

COR: crude odd ratio, AOR: adjusted odds ratio.

## Data Availability

The dataset used in this study is available from the corresponding authors on a reasonable request.
